# Effect of Dibutyltin Dilaurate on Triglyceride Metabolism through the Inhibition of the mTOR Pathway in Human HL7702 Liver Cells

**DOI:** 10.3390/molecules23071654

**Published:** 2018-07-06

**Authors:** Xiaozhi Qiao, Yunlan Li, Jiaqi Mai, Xiaoqing Ji, Qingshan Li

**Affiliations:** 1 School of Pharmaceutical Science, Shanxi Medical University, Taiyuan 030001, China; 18734103763@163.com (X.Q.); 18734123615@163.com (J.M.); 18435147978@163.com (X.J.); 2 Department of Traditional Chinese Medicine, Shanxi University of Traditional Chinese Medicine, Jinzhong 030619, China

**Keywords:** dibutyltin dilaurate (DBTD), triglycerides metabolism, mTOR pathway, SREBP1C, PPARα

## Abstract

Dibutyltin dilaurate (DBTD) has multiple applications in daily life. However, DBTD is easily deposited in the liver and affects liver functions. This study was designed to explore the effects of DBTD on triglyceride metabolism in human normal hepatocyte HL7702 cells. Our results showed that the intracellular fat contents were dose-dependently decreased by DBTD. The expression of lipolysis genes and proteins were elevated while the lipogenesis genes and proteins were diminished by DBTD. The phosphorylation levels of ribosomal S6 kinase 1 were reduced by both rapamycin and DBTD, indicating that the mTOR pathway was suppressed possibly. The decreased sterol regulatory element-binding protein 1C (SREBP1C) transcription levels, as well as the increased peroxisome proliferator-activated receptor alpha (PPARα) transcription levels, caused by rapamycin and DBTD corresponded to the inactive mTOR pathway. In conclusion, it was possible that DBTD reduced the intracellular triglyceride through depressing the mTOR pathway and affecting its downstream transcription factors.

## 1. Introduction

Dibutyltin compounds (DBTs), for example dibutyltin dilaurate (DBTD) and dibutyltin dichloride (DBTC), consist of two butyls that have covalently bonded with an Sn (IV). DBTs are closely related with our daily life and are widely used as polyvinyl chloride (PVC) plastic stabilizers, pesticides, marine antifouling coatings, and catalysts in rubber, polyurethane (PU), and the biodiesel industry [1,2,3]. DBTs can also be employed as catalysts in the production of biological materials, such as blood-contacting biomaterials and scaffolds for bone tissue engineering [4,5].

Unfortunately, lots of studies have shown that DBTs are able to accumulate in and even contaminate the aquatic environment as well as cultivated land and remain unchanged for years [6,7]. People could uptake DBTs from eating and drinking. Jesper B. Nielsen et al. measured organotins in livers of Danish men, and found that DBTs appear to be the main species deposited in the liver [8].

The liver is the main organ for metabolism where fatty acid oxidation and lipogenesis proceed. Early studies reported that DBTs treatment led to liver and bile duct lesions in rodents [9,10]. It was recently reported that, after treatment with DBTC, tumor necrosis factor receptors 1 and 2 (*TNFR1/R2*)-deficient mice showed liver fatty degeneration [11]. However, the effect of DBTs on triglyceride (TG) metabolism in normal liver cells has seldom been reported on.

Our previous proteomics research on rat livers had found that DBTs mainly affected the proteins related to lipid anabolism and catabolism [12]. The TG metabolism in the liver is regulated by the mammalian target of rapamycin (mTOR) pathway through modulating peroxisome proliferator-activated receptor alpha (PPARα) and sterol regulatory element-binding protein 1C (SREBP1C) [13,14]. There are three PPAR isoforms (α, γ, and β/δ) and they are have different distributions and functions as transcription factors. PPARγ is abundant in adipose tissue and associated with adipocyte differentiation. PPARα, predominantly expressed in the liver and kidney, is able to regulate mitochondrion and peroxisome fatty acid beta oxidation as well as microsomal omega oxidation by promoting the transcription of genes that contain the peroxisome proliferation responsive element (PPRE) sequence, for example PPARα, Acyl-CoA oxidase 1 (ACOX1), Carnitine palmitoyltransferase I (CPT1), and Cytochrome P450 4A11 (CYP4A11) [15]. ACOX1 is involved in peroxisomal fatty acid beta oxidation. CPT1 is a rate-limit enzyme for peroxisomal fatty acid β oxidation. In microsomes, long-chain fatty acids undergo ω oxidation and are catalyzed by CYP4A11 into dicarboxylic acids, which will be further metabolized through β oxidation [16]. Excessive accumulation of dicarboxylic acids would lead to cell damage. PPARα are commonly inactivated and combined with a corepressor, such as nuclear receptor co-repressor 1 (NCoR1). Once activated by their ligands, PPARα could dimerize with retinoid X receptor (RXR), recruit coactivators, and release a corepressor to induce the transcription of genes involved in fatty acid oxidation [17]. mTORC1 facilitates the accumulation of NCoR1 in the nucleus to depress the transcription of PPARα and fatty acid oxidation [18].

SREBP1C is an essential transcription factor that regulates TG synthesis. There are three SREBP isoforms, among which SREBP1C is the main transcription regulator for lipogenic genes [19]. In endoplasmic reticulum, the inactive SREBP1C precursor (pre-SREBP1C) binds with SREBP cleavage-activating protein (SCAP) into a complex, which can be cleaved in Golgi to facilitate their entrance into the nucleus [20]. The mature SREBP1C bind to the sterol regulatory elements (SREs) of target genes, such as SREBP1C, stearoyl-CoA desaturase 1 (SCD1), fatty acid synthase (FASN), and glycerol-3-phosphate acyltransferase (GPAT1). It is required that SREBP1C dimerizes with another bHLH protein, such as SREBP1C or upstream stimulatory factor 1 (USF1), to promote transcription of target genes. SCD1 and FASN are enzymes that play important roles in fatty acid synthesis; SCD1 is a rate-limiting enzyme in monounsaturated fatty acid biosynthesis, and FASN catalyzes the lipogenic steps of converting acetyl-CoA and malonyl-CoA into palmitate. GPAT1 catalyzes the first step of triglyceride formation. The activities of SREBP1C are modulated by mTORC1 through the activation of S6 kinase (S6K1), which not only promotes mature SREBP1C but also elevates their phosphorylation [21]. The aim of the present research was to explore the effect of DBTD on TG metabolism and detect whether mTORC1 is involved in it.

## 2. Results

### 2.1. DBTD Decreases the Viability of Liver Cells

**Figure 1:**
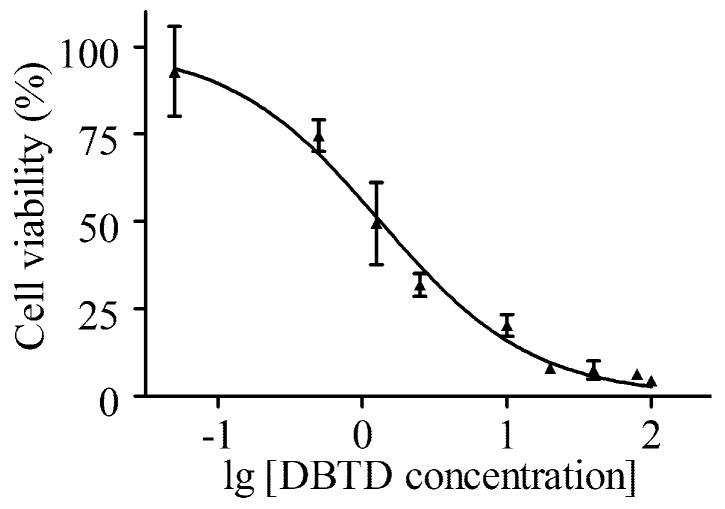
Toxicity of dibutyltin dilaurate (DBTD) on the HL7702 cell line. Cells were treated with a series of diluted DBTD (100, 80, 40, 20, 10, 5, 2.5, 1.25, 0.50, 0.05, and 0 μmol/L) for 24 h. Data were obtained from three independent experiments (*n* = 3). Values are mean ± SD.

An MTT assay was applied to test the effect of DBTD on HL7702 cells viability. This assay is based on the detection of succinate dehydrogenase (SDH) in living cells and is commonly applied to determine IC_50_, which is helpful in estimating the toxicity of exogenous agents and providing a reference for a dose range [22]. Figure 1 shows the HL7702 cell viability curve; the IC_50_ for treatment with DBTD for 24 h was 1.33 μmol/L. Considering that people are seldom in contact with high doses of organotin in day-to-day life, 0, 0.13, 0.25, 0.50, 1.00, and 2.00 μmol/L DBTD were set in the following studies.

### 2.2. Activities of Alanine Aminotransferase (ALT) and Aspartate Aminotransferase (AST) Are Elevated in the Cultural HL7702 Cell Media by DBTD

Once hepatocytes are damaged, ALT and AST will be released out of cells into the culture media. ALT and AST are frequently used as laboratory biomarkers of hepatotoxicity due to the fact that they catalyze alanine or aspartate, respectively, in the presence of alpha-ketoglutarate into pyruvate, which can be detected to calculate the activities of ALT and AST [23]. It has been reported that DBTs could cause liver damage and increase the ALT and AST activities in the blood of rodents [24]. Herein, the activities of ALT and AST in the culture media were tested by ALT and AST kits, respectively. The results showed that both ALT and AST had significant dose-dependent increases (*p* < 0.05) in 0.50, 1.00, and 2.00 μmol/L groups compared to the control group (Table 1).

### 2.3. The Intracellular TG Contents Visualized by Oil-Red O Staining Were Reduced

**Figure 2:**
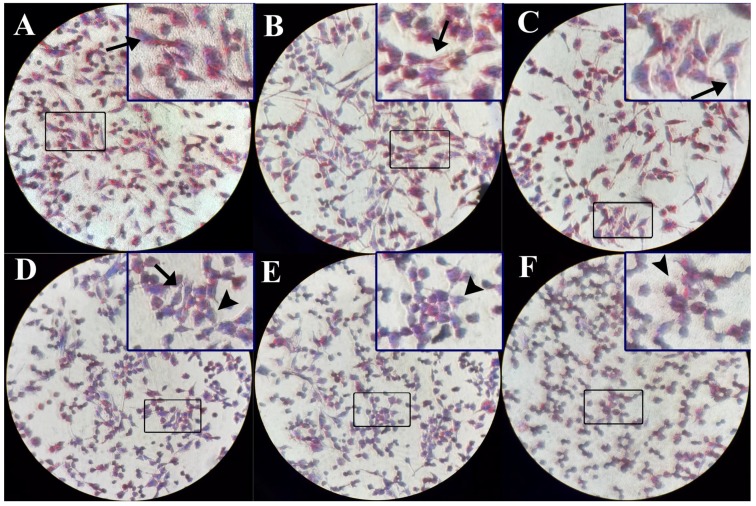
Intracellular triglyceride (TG) stained by oil-red o (ORO) after treatment with different concentrations of DBTD. The ORO kits stain the lipid red and nucleus blue, respectively, to facilitate our observation of lipid droplets. (**A**) 0 μmol/L, (**B**) 0.13 μmol/L, and (**C**) 0.25 μmol/L DBTD-treated cells have large red stained sections and have normal morphology stretching in fibrous shapes (arrow). (**D**) 0.50 μmol/L, (**E**) 1.00 μmol/L, and (**F**) 2.00 μmol/L DBTD-treated cells have diminished red areas and turn to a global appearance, which indicates low viability (arrow head). Original magnification ×100.

Liver cells were incubated with 0, 0.13, 0.25, 0.50, 1.00, and 2.00 μmol/L of DBTD. We visualized the intracellular TG by oil-red o (ORO) staining. As is shown in Figure 2 and Table 2, TG accumulations in HL7702 cells were decreased in the 0.50, 1.00, and 2.00 μmol/L groups in contrast to the control group. Moreover, we noticed that the morphology of cells changed from fibrous in the control group to global in those groups. Our findings indicated that treatment with DBTD caused a TG decrease and cell lesions.

### 2.4. The TG Contents Were Dose-Dependently Decreased by DBTD in Liver Cells

**Figure 3:**
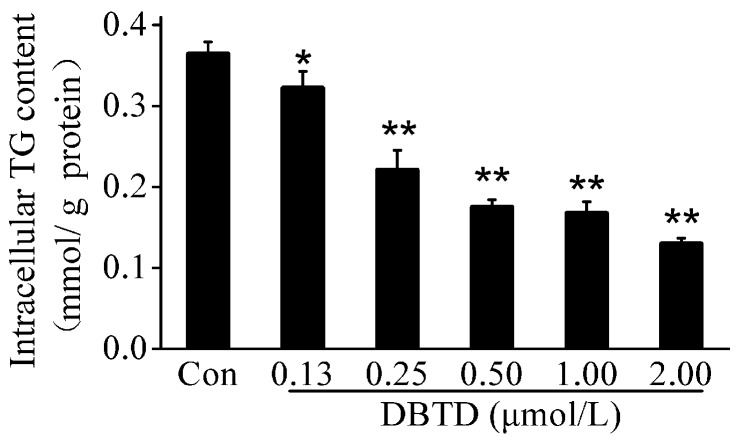
Triglyceride (TG) contents in HL7702 cells treated with different concentrations of DBTD (0, 0.13, 0.25, 0.50, 1.00, and 2.00 μmol/L) for 24 h. The TG concentrations were normalized to the corresponding total cell protein contents. Values are expressed as the mean ± SD (*n* = 3), * *p* < 0.05, ** *p* < 0.01 versus control. Con = control.

The TG contents were measured by TG kits to further verify whether DBTD reduced the TG contents and clarify the relationship between DBTD dosage and TG contents in hepatocytes. After cells were treated with 0, 0.13, 0.25, 0.50, 1.00, and 2.00 μmol/L DBTD, there were significant (*p* < 0.05) concentration-dependent decreases of the intracellular TG, which varied from 0.36 ± 0.01 to 0.13 ± 0.01 mmol/g protein (Figure 3).

### 2.5. Exposure to DBTD Leads to Low Expression of Lipogenic Genes and High Expression of Lipolytic Genes in Liver Cells

**Figure 4:**
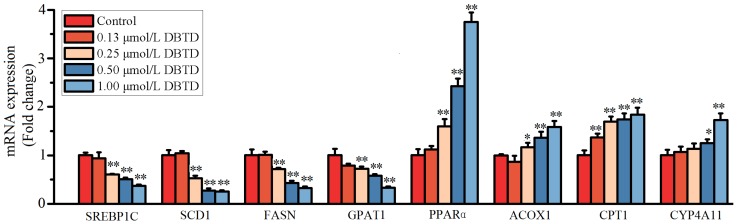
Fold changes of genes involved in triglyceride (TG) metabolism after treatment with 0, 0.13, 0.25, 0.50, and 1.00 μmol/L DBTD for 24 h in contrast to the control group (Con). The expressions of lipogenic genes, including *SREBP1C*, *SCD1*, FASN, and *GPAT1*, are depressed by DBTD. The expressions of *PPARα, ACOX1, CPT1*, and *CYP4A111* that involved in fatty acid oxidation are induced by DBTD. The data are expressed as the mean ± SD (*n* = 3), * *p* < 0.05 versus control, ** *p* < 0.01 versus control.

To analyze the way DBTD reduced the intracellular TG, we performed RT-PCR to measure the expression of genes involved in TG metabolism. Our results showed that the relative expression of *SREBP1C*, *SCD1, FASN*, and *GPAT1*, which regulate the biosynthesis of TG, was significantly (*p* < 0.05) downregulated by 0.25, 0.50, and 1.00 μmol/L DBTD, respectively, in contrast to the control (Figure 4). The expression of *PPARα, ACOX1*, and *CPT1*, which take part in the fatty acid β oxidation, was significantly (*p* < 0.05) upregulated by 0.25, 0.50, and 1.00 μmol/L DBTD, respectively, in contrast to the control (Figure 4). The expression of *CYP4A11* was unchanged at concentrations of DBTD lower than 0.5 μmol/L, while it was significantly increased at 0.5 and 1.0 μmol/L DBTD (1.74-fold and 1.83-fold, respectively) (*p* < 0.05), which indicated that omega oxidation might not be the main cause for TG degradation when cells are treated with a low dosage of DBTD. These results indicated that DBTD may decrease the TG content through downregulating lipogenesis genes and upregulating the genes responsible for fatty acid oxidation.

### 2.6. Lipogenic Proteins Are Decreased and Lipolytic Proteins Are Increased in DBTD-Treated Liver Cells

**Figure 5:**
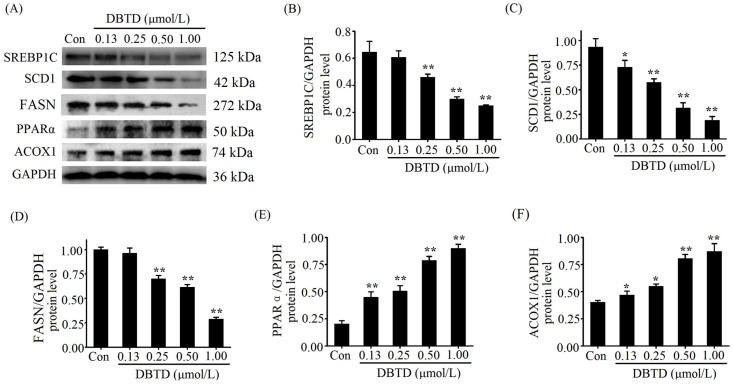
Western blot analysis of SREBP1C, SCD1, FASN, PPARα, and ACOX1 in HL7702 cells treated with 0, 0.13, 0.25, 0.50, and 1.00 μmol/L DBTD for 24 h. (**A**) Representative bands of relevant proteins. Relative protein levels of (**B**) SREBP1C, (**C**) SCD1, (**D**) FASN, (**E**) PPARα, and (**F**) ACOX1 in liver cells treated with DBTD. Quantities are expressed as relative intensities against GAPDH. Data are presented as the mean ± SD of three experiments. * *p* < 0.05 and ** *p* < 0.01 versus control.

SREBP1C and PPARα are transcription factors, while SCD1, FASN, and ACOX1 are enzymes that participate in TG metabolism and their chemical essence is proteins. To identify the impact of DBTD on lipid metabolism, we examined the related protein levels in hepatocytes after treatment with different concentrations of DBTD. Figure 5 shows the immunoblot bands and quantified values. The protein levels of SREBP1C and FASN were significantly (*p* < 0.05) decreased in the 0.25, 0.50, and 1.00 μmol/L DBTD groups compared to the control (Figure 5B,D). The levels of SCD1 were significantly (*p* < 0.05) decreased in a dose-dependent manner by DBTD (Figure 5C). PPARα and ACOX1 were significantly increased in all DBTD-treated cells (*p* < 0.05) (Figure 5E,F). Those results threw light on our original assumption and verified that DBTD can inhibit lipid formation and induce TG degradation.

### 2.7. DBTD Affects TG Metabolism through Inhibiting the mTOR Pathway

**Figure 6:**
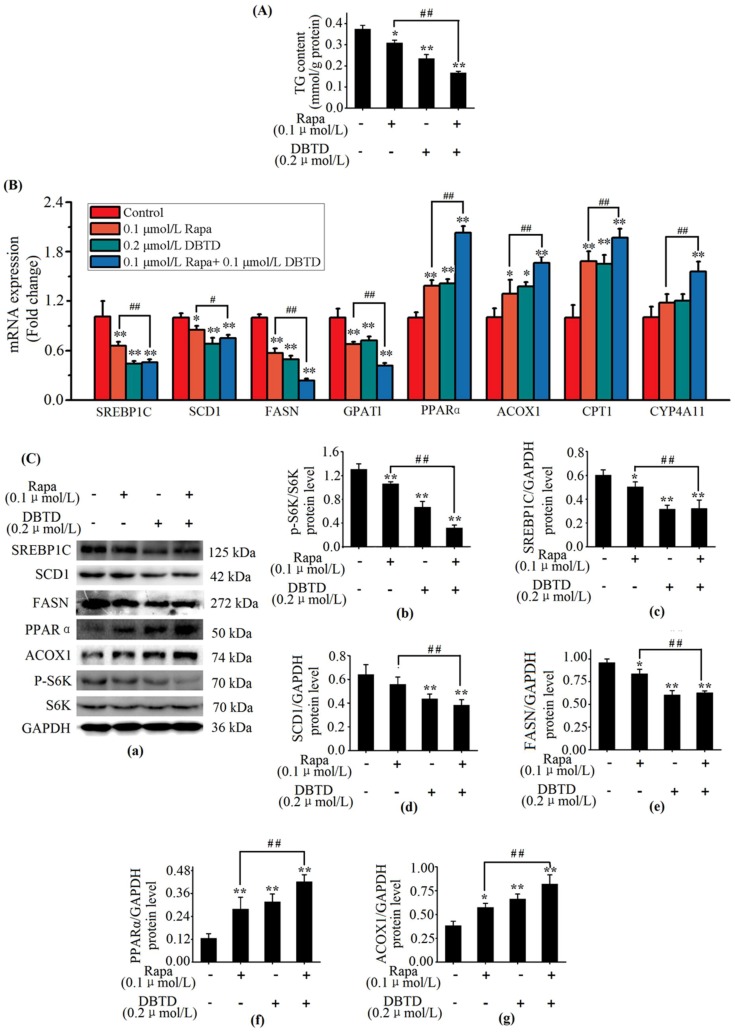
Effects of DBTD on the mTOR pathway and triglyceride (TG) metabolism. Cells were treated with 0.1 μmol/L rapamycin (Rapa) for 6 h, 0.2 μmol/L DBTD for 24 h, and pretreated with 0.1 μmol/L Rapa for 6 h followed by a 24 h treatment of 0.2 μmol/L DBTD, respectively. The control group (Con) was incubated with culture media only. (**A**) measurement of intracellular TG content. (**B**) Expressions analysis of *SREBP1C*, *SCD1*, *FASN*, *GPAT1*, *PPARα*, *ACOX1, CPT1*, and *CYP4A11* by RT-PCR. (**C**) Protein analysis by immunoblot, (**a**) representative bands of relevant proteins. Protein levels of (**b**) p-S6K1 (Thr421), (**c**) SREBP1C, (**d**) SCD1, (**e**) FASN, (**f**) PPARα, and (**g**) ACOX1. Quantities are expressed as relative intensities against GAPDH except for p-S6K1 (Thr421), which was against S6K1. Data are presented as the mean ± SD of three experiments. * *p* < 0.05 and ** *p* < 0.01 versus control. # *p* < 0.05 and ## *p* < 0.01 versus Rapa group.

The metabolism in liver cells was modulated by the mTOR pathway. Hepatocytes with a blocked mTOR pathway had decreased intracellular TG contents as well as activated PPARα and depressed SREBP1C transcription activities [25]. Rapamycin is a specific inhibitor of mTORC1 which can increase lipolysis and decrease lipogenesis in mice livers [26]. In order to explore whether SREBP1C and PPARα in HL7702 were modulated through the mTOR pathway by DBTD, we treated cells with rapamycin and DBTD. Considering that damage to cells could result in a decrease of lipogenic genes and p-S6K1 protein, we treated cells with a safe dosage of DBTD (0.2 μmol/L) and rapamycin (0.1 μmol/L) in this experiment; i.e., a dosage at which the activities of ALT and AST in the culture media and cell viability were not affected (Table 3 and Table 4). Then, we measured the intracellular TG with a TG kit and performed RT-PCR as well as Western blot to contrast the effect of 0.2 μmol/L DBTD with 0.1 μmol/L rapamycin on TG metabolism. When DBTD and rapamycin were given separately, the TG contents in both groups (0.23 ± 0.02 and 0.31 ± 0.01 mmol/g protein, respectively) were significantly decreased in contrast to the non-treated group (0.38 ± 0.02 mmol/g protein) (*p* < 0.05) (Figure 6A). Rapamycin is able to inhibit the mTOR pathway and bring down the phosphorylation state of S6K1, which is a downstream effector as well as an admitted indicator for mTORC1 activity [27]. Our results showed that the phosphorylation level of S6K1 in both the DBTD and rapamycin groups was decreased from 1.31 ± 0.09 to 0.67 ± 0.10 and 1.06 ± 0.04, respectively (Figure 6C(b)). Gene expressions of *SREBP1C*, *SCD1, FASN*, and *GPAT1* (Figure 6B) as well as the protein levels of SREBP1C and FASN (Figure 6C(c,e)) were significantly depressed (*p* < 0.05). Gene expressions of *PPARα*, *ACOX1*, and *CPT1* as well as the protein levels of PPARα and ACOX1 (Figure 6B,C(f,g)) exhibited contrary changes in the DBTD and rapamycin groups compared to the blank group. Our results indicated that DBTD is a depressor of the mTOR pathway and affects the TG metabolism through inhibiting lipogenesis and activing fatty acid β oxidation in liver cells.

In present study, 0.1 μmol/L rapamycin inhibited the mTOR pathway and the addition of 0.2 μmol/L DBTD lead to further inhibition. Concretely, cells treated with DBTD following rapamycin had a lower TG content and p-S6K1 (Thr421) protein level in contrast to rapamycin-treated cells (0.17 ± 0.01 versus 0.31 ± 0.01 mmol/g protein and 0.32 ± 0.05 versus 1.06 ± 0.04, respectively) (Figure 6A,C(a,b)). Gene analysis showed that the expressions of *SREBP1C*, *SCD1, FASN*, and *GPAT1* were significantly (*p* < 0.05) decreased by 30%, 12%, 58%, and 38% (0.66 ± 0.05 versus 0.46 ± 0.04, 0.85 ± 0.04 versus 0.75 ± 0.04, 0.57 ± 0.05 versus 0.24 ± 0.02, and 0.68 ± 0.03 versus 0.42 ± 0.03) in the rapamycin coupled with DBTD treatment group compared to the rapamycin treatment group, respectively (Figure 6B). These changes are coincident with the changes in p-S6K1 (Thr421) protein level, which was decreased by 70% (0.32 ± 0.05 versus 1.06 ± 0.04) (Figure 6C(b)). On the contrary, the transcription levels of *PPARα, ACOX1*, *CPT1*, and *CYP4A11* were significantly (*p* < 0.05) increased by 46%, 29%, 17%, and 41% (1.39 ± 0.07 versus 2.03 ± 0.08, 1.29 ± 0.17 versus 1.66 ± 0.08, 1.68 ± 0.12 versus 1.97 ± 0.11, and 1.19 ± 0.13 versus 1.68 ± 0.12, respectively, compared to the control) (Figure 6B), which were adverse to the change in p-S6K1 (Thr421) level (Figure 6C(b)). Our results further indicated that mitochondrial and peroxisomal fatty acid β oxidation as well as microsomal ω oxidation were activated by DBTD while lipogenesis was depressed by DBTD (Figure 6). We speculated that DBTD might be a depressor of the mTOR pathway, and DBTD affected TG metabolism through SREBP1C and PPARα by inhibiting the mTOR pathway.

## 3. Discussion

Mountains of research have been reported that DBTs are hepatotoxic [28,29]. Our research showed that HL7702 cells were damaged by DBTD at concentrations higher than 0.5 μmol/L, which was confirmed by increased ALT and AST activities in the culture media and an abnormally changed cell morphology (Table 1, Figure 2). Additionally, we found that 0.5 μmol/L to 2.0 μmol/L DBTD reduced the TG contents in liver cells in a dose-dependent manner (Figure 2 and Figure 3, Table 2). To our surprise, there were hardly any reports exploring the effect of DBTD on liver TG metabolism. In the study of Flora A. Milton [30], DBTs promoted 3T3-L1 preadipocytes differentiation and adipogenesis through activating retinoid X receptor α (RXRα) and PPARγ. Due to the fact that DBTD is a partial PPARγ and RXRα agonist [31], and, moreover, that PPARγ has little distribution in the liver, we presumed that DBTD might have different effects on TG metabolism between the liver and fatty tissues.

**Figure 7:**
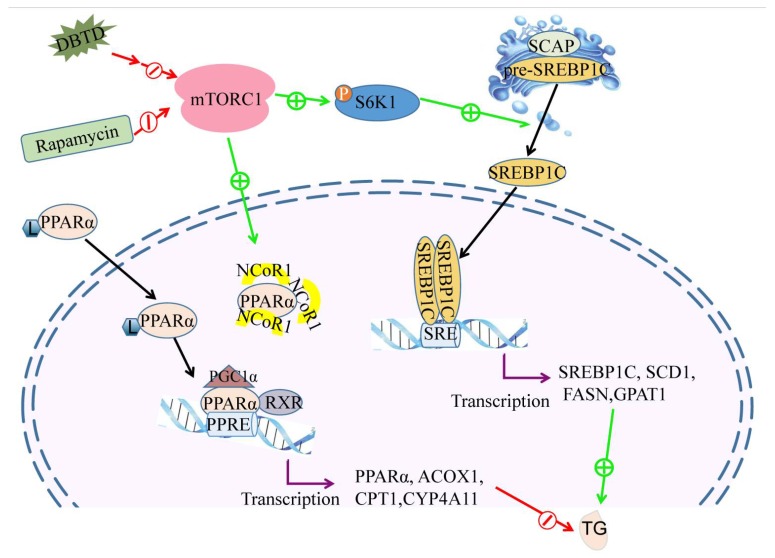
DBTD decreases the intracellular TG by promoting fatty acid β oxidation and inhibiting lipogenesis through blocking the mTOR pathway. Fatty acid oxidation is regulated by PPARα. Once activated by their ligands (L), PPARα could dimerize with RXR, recruit coactivators (PGC1α), and release a corepressor (NCoR1) to bind the PPRE of lipolysis genes, such as PPARα, ACOX1, CPT1, and CYP4A11, to activate their transcription. mTORC1 facilitates the accumulation of NCoR1 in the nucleus to depress the transcription of PPARα and inhibit fatty acid oxidation. Lipogenesis are regulated by SREBP1C. In endoplasmic reticulum, the inactive SREBP1C precursor (pre-SREBP1C) binds with SREBP cleavage-activating protein (SCAP) into a complex, which can be cleaved in Golgi to facilitate their entrance into the nucleus. The mature SREBP1C binds to the sterol regulatory elements (SREs) of target genes, such as SREBP1C, SCD1, FASN, and GPAT1. It is required that SREBP1C dimerize with another bHLH protein, such as SREBP1C and upstream stimulatory factor 1 (USF1), to promote transcription of target genes. Rapamycin and DBTD depress the activity of mTORC1, so the transcription of SREBP1C is reduced while the transcription of PPARα is enhanced, which will lead to a TG content decrease.

Our previous research found that the gene expressions of platelet glycoprotein 4 (*CD36*), fatty acid binding protein (*FABP*), enoyl-CoA hydratase (*EHHADH*), and 3-ketoacyl-CoA thiolase B (*ACAA1*), which are involved in fatty acid β oxidation, were increased in Buffalo Rat liver cells (BRL) treated with DBT and Di-*n*-butyl-di-(4-chlorobenzohydroxamato) tin (IV) (DBDCT) [12]. Our present research showed that the TG contents as well as the genes involved in lipogenesis, including *SREBP1C, SCD1*, *FASN,* and *GPAT1,* were dosage-dependently decreased when cells were treated with DBTD at concentrations higher than 0.25 μmol/L (Figure 3 and Figure 4), while the gene expressions of *PPARα*, *ACOX1*, and *CPT1* in HL7702 cells were increased (Figure 4). The expression of *CYP4A11* was upregulated by 0.5 and 1.0 μmol/L DBTD. Our results indicated that the SREBP1C pathway and the PPARα pathway are responsible for the abnormal TG metabolism in liver cells caused by DBTD (Figure 7). Additionally, fatty acid ω oxidation might take less of a role in fat degradation than β oxidation at lower concentration of DBTD.

There is a lack of reports on the relationship between DBTs and PPARα; however, it was reported that DBTs were partial ligands of PPARγ that could bind with PPARγ directly and showed weak agonistic activity [31]. In view of the fact that the activating ligands of PPARs are semiselective for the subtypes (α, β, γ) and rely on ligand concentration and cell type [17], PPARα might be directly activated by DBTD as an activating ligand or indirectly activated by DBTs through mTORC1. mTORC1 is of great importance in modulating the TG metabolism in response to insulin, nutrition, and exogenous objects [32,33]. Considering that both PPARα and SREBP1C are downstream effectors of mTORC1 and are affected by DBTD [18,19], it was most likely that the mTOR pathway was involved in the action of DBTD on TG metabolism. mTORC1 consists of mTOR, Raptor, mammalian ortholog of LST8 (mLST8), PRAS40, and Deptor. The activity of mTORC1 is dependent on its structure and phosphorylation level. The readouts of mTORC1, including its intrinsic kinase activity and downstream phosphorylation level, are of S6K1 [27]. Because rapamycin inhibits the activity of mTORC1 through blocking the interaction between mTOR and Raptor without disturbing the autophosphorylation activity and the mechanism of action between DBTD and mTORC1 is unclear [34], the p-S6K1 (Thr421) levels were applied in the present study to measure the activity of mTORC1 affected by rapamycin and DBTD. Our results showed that the levels of p-S6K1 (Thr421) were decreased by DBTD and rapamycin without damaging cells’ integrity (Figure 6C(a,b), Table 3 and Table 4). In the meantime, the activities of the downstream effectors of mTOR were affected accordingly. Concretely, the gene expressions of *PPARα*, *ACOX1*, and *CPT1* were upregulated, while the expressions of *SREBP1C, SCD1, FASN*, and *GPAT1* were downregulated (Figure 6B) by DBTD and rapamycin. The activity of mTORC1 is affected by growth factors, energy status, cell apoptosis, and necrosis. Our present research was not enough to point out the particular target in the mTOR pathway of DBTD and the particular mechanism of the inactivated mTORC1 by DBTD. We speculated that the mTOR pathway was involved in DBTD’s regulation of TG metabolism (Figure 7). The changes in protein levels of PPARα, ACOX1, SREBP1C, and SCD1 also supported our speculation (Figure 6C).

## 4. Materials and Methods

### 4.1. Reagent

DBTD was purchased from J&K Scientific Ltd (512404, Beijing, China). Methyl thiazolyl tetrazolium (MTT, M8180, Solarbio, Beijing, China) was dissolved with PBS to 5 mg/mL prior to use. Aspartate aminotransferase (AST), alanine aminotransferase (ALT), TG, and oil-red o (ORO) staining assay kits were from Nanjing Jiancheng bioengineering Institute (Nanjing, China). HL-7702 cells (CX0157) and cell culture media RPMI 1640 were obtained from Boster Biological Engineering Co., Ltd (Wuhan, China). Fetal bovine serum (FBS) was from Tianhang Biotechnology Co., Ltd (Hangzhou, Zhejiang, China). The agents for RT-PCR were from TakaRa Biomedical Technology Co., Ltd (Beijing, China). Primers were designed and purchased from Sangon Biotech Co., Ltd (Shanghai, China). Primary antibodies were SREBP1C (14088-1-AP, Proteintech), SCD1 (BS60618, Bioworld), FASN (10624-2-AP, Proteintech), PPARα (BZ04355, Bioworld), ACOX1 (BZ05292, Bioworld), S6K1 (BS1280, Bioworld), p-S6K1 (Thr421) (BS4853, Bioworld), and GAPDH (AP0063, Bioworld). The second antibody was goat anti-rabbit IgG (BA1054, Boster), and the marker was purchased from Mei5 Biotechnology Co., Ltd (MF028-plus, Beijing, China). The rapamycin (Rapa, aladdin) stock solutions were dissolved in DMSO to 1 mmol/L.

### 4.2. Cell Culture and Treatment

HL-7702 cells were grown in RPIM 1640 supplemented with 10% fetal bovine serum and maintained in a humidified CO_2_-regulated incubator with a 5% CO_2_ atmosphere at 37 °C (Heal Force, HF90). DBTD was dissolved in DMSO to form the stock solutions at a concentration of 10 mmol/L. The rapamycin stock solutions were dissolved in DMSO to 10 mmol/L. To inhibit the activity of the mTOR pathway, cells were treated with 0.1 μmol/L rapamycin for 6 h. To observe the impact of DBTD on cells pretreated with rapamycin, HL7702 cells were pretreated with 0.1 μmol/L rapamycin for 6 h followed by a treatment of 0.2 μmol/L DBTD for 24 h.

### 4.3. Viability of Cells

For the viability assay, HL-7702 cells were seeded in 96-well plates. When the density reached 1 × 10^6^ cells per well, DBTD (100, 80, 40, 20, 10, 5, 2.5, 1.25, 0.50, and 0.05 μmol/L) was added. After 24 h of treatment, the medium was removed and the cells were washed with PBS, and 100 μL FBS-free medium and 10 μL MTT solution (5 mg/mL in PBS) were added to each well. The plates were incubated for 4 h to allow for the formation of purple formazan crystals, and the supernatant was carefully removed so as not to disturb the formazan crystals. Then, 100 μL DMSO was added to solubilize the crystals and the absorbance was read at 570 nm or 490 nm in an automatic microplate spectrophotometer (Thermo Scientific, Waltham, MA, USA). The IC_50_ was calculated using graphpad prism 5 software (5.01, GraphPad Software, San Diego, CA, USA). The viability of cells treated with rapamycin was determined according to the approach above.

### 4.4. Activities of ALT and AST and Intracellular TG Contents Measurement

HL-7702 cells were seeded in a 6-well plate, the control group was treated with medium only, and the experimental groups were treated with a series of DBTD concentrations of 0.13, 0.25, 0.50, 1.00, and 2.00 μmol/L for 24 h. The media of each group were collected to measure the activities of AST and ALT using activity determination kits, respectively, according to the manufacturer’s instructions. Cells were washed with PBS and collected with a scraper to measure the intracellular lipid content by using a TG determination kit. The TG concentrations were normalized to the total cell protein concentrations. The activity of the ALT and AST of cells treated with rapamycin was determined according to the procedure above.

### 4.5. Oil Red O Staining

HL-7702 cells were grown on a coverslip, and they were treated with different concentrations of DBTD (0, 0.13, 0.25, 0.50, 1.00, and 2.00 μmol/L) for 24 h when they became stuck and had grown to a suitable density. The medium was removed, and the cells were washed with PBS then fixed with 4% paraformaldehyde solution for 15 min at room temperature. After fixation, cells were washed gently with PBS and stained with oil red o according to the manufacturer’s instructions. The intracellular lipids were observed under the microscope (BDS200-PH, OPTEC, Chongqing, China) and pictures were taken. The cells were gently washed with 60% isopropanol, and 500 μL isopropanol was added to dissolve the oil red-o dye in cells. The absorbance was detected at 492 nm [35].

### 4.6. Quantitative Real-Time PCR

The total RNA of HL-7702 cells was isolated using total RNA extraction kits according to the manufacturer’s instructions. The purity and the concentration of RNA were detected by an automatic microplate spectrophotometer (Thermo Scientific, USA). Then, 1 µg of total RNA was used for the preparation of cDNA using a kit following the manufacturer’s instructions. An SYBR kit was used to perform the amplification step on a StepOnePlus^TM^ PCR System. The reaction mixture underwent 95 °C for 30 s followed by 45 cycles of 95 °C for 5 s and 60 °C for 30 s. The system also ran a melting curve reaction to verify the specificity of the PCR products. The relative levels of the target genes were determined by the 2^−∆∆CT^ method with GAPDH as a reference gene. The data were presented as the fold changes over the control group, and the primers are shown in Table 5.

### 4.7. Western Blot Analyses

After being treated, cells were collected to extract total protein with a radioimmuno precipitation assay (RIPA). Lysisas, protease, and phosphatase inhibitors should be added before use. An ultrasonic cell disrupter (VCX 130, Sonics & Materials Inc., Newtown, CT, USA) was used to homogenize cells on ice, then the homogenates were centrifuged (13,000 r·min^−1^, 15 min, 4 °C) and the supernatant was taken as a total protein solution. The concentration of each group was determined by a BCA protein assay kit. Forty microgram (40 μg) protein samples were loaded in the lanes to perform sodium dodecyl sulfate­polyacrylamide gel electrophoresis (SDS­PAGE) and the separated proteins were electro-transferred onto a nitrocellulose membrane (221BR 46606, BIO-RAD, Hercules, CA, USA). The membrane was shaken with 5% non-fat milk for 1 h at room temperature to block nonspecific binding sites. Next, the membrane was incubated with the corresponding primary antibodies at 4 °C overnight. After being washed with TBST three times, the membranes were then incubated with secondary anti IgG monoclonal antibody for 1 h at room temperature. Finally, the membranes were washed with TBST three times. Specific bands were detected using an enhanced chemiluminescence kit, and the gray level was quantified using photoshop CS5 software (Adobe, San Jose, CA, USA). The protein levels were normalized against GAPDH.

### 4.8. Statistical Analyses

All experiments were repeated at least three times. Values are shown as mean ± SD. The significance of differences was determined by one-way ANOVA using the SPSS 22.0 software (SPSS, Chicago, IL, USA). A value of *p* < 0.05 was considered statistically significant.

## 5. Conclusions

This study explored the effect of DBTD on liver cells from the two aspects of TG metabolism, namely synthesis and degradation. The current results showed that DBTD can decrease the intracellular TG notably through blocking the mTOR pathway. On the one hand, DBTD promotes *PPARα* transcription and fatty acid oxidation, and on the other hand, it inhibits *SREBP1C* transcription and lipogenesis (Figure 7). However, this study was not enough to elucidate the inhibitory mechanism of DBTD on the mTOR pathway. Additionally, the impacts of long-term exposure to DBTD on liver TG in a whole-body context are still unclear. Further research needs to be conducted to fully learn the effect on liver TG caused by DBTD.

## Figures and Tables

**Table 1:** Activities of alanine aminotransferase (ALT) and aspartate aminotransferase (AST) in the culture media measured by kits after treatment with different concentrations of DBTD for 24 h.

DBTD (μmol/L)						
	0	0.13	0.25	0.50	1.00	2.00
ALT (IU/L)	0.93 ± 0.69	0.86 ± 0.51	1.52 ± 1.00	2.41 ± 1.58 *	3.99 ± 0.32 **	5.45 ± 0.44 **
AST (IU/L)	2.94 ± 1.89	2.26 ± 1.45	4.41 ± 0.65	5.77 ± 1.13 *	9.72 ± 0.84 **	10.51 ± 1.37 **

Notice: Data were obtained from three independent experiments (*n* = 3). Values are mean ± SD, * *p* < 0.05, ** *p* < 0.01 versus the control.

**Table 2:** The absorbance of oil-red o dye dissolved in isopropanol.

	DBTD (μmol/L)					
	0	0.13	0.25	0.5	1.0	2.0
Absorbance	0.194 ± 0.008	0.193 ± 0.004	0.188 ± 0.006	0.156 ± 0.007 **	0.136 ± 0.006 **	0.102 ± 0.006 **

Notice: Data were obtained from three independent experiments (*n* = 3). Values are mean ± SD, ** *p* < 0.01 versus the control.

**Table 3:** Viability of cells treated with DBTD and rapamycin.

Group	Blank Group	Rapa (0.1 μmol/L)	DBTD (0.2 μmol/L)	Rapa (0.1 μmol/L) + DBTD (0.2 μmol/L)
Viability (%)	100	102 ± 11	93 ± 8	98 ± 5

Notice: Data were obtained from three independent experiments (*n* = 3). Values are mean ± SD, *p* > 0.05, versus blank group. Rapa = rapamycin.

**Table 4:** Activities of ALT and AST in the culture media measured by kits after treatment with DBTD and rapamycin.

Group	ALT (IU/L)	AST (IU/L)
Blank group	0.91 ± 0.61	2.83 ± 0.58
Rapa (0.1 μmol/L)	1.05 ± 0.39	2.77 ± 0.2
DBTD (0.2 μmol/L)	1.01 ± 0.59	2.79 ± 0.52
Rapa (0.1 μmol/L) + DBTD (0.2 μmol/L)	1.06 ± 0.47	2.83 ± 0.52

Notice: Data were obtained from three independent experiments (*n* = 3). Values are mean ± SD, *p* > 0.05 versus blank group. Rapa = rapamycin.

**Table 5:** Primers for RT-PCR.

Genes	Primer Sequence (5′-3′)		Length (bp)
*SREBP1C*	Forward	CGGAACCATCTTGGCAACAGT	141
	Reverse	CGCTTCTCAATGGCGTTGT	
*SCD1*	Forward	TTCCCGACGTGGCTTTTTCT	149
	Reverse	AGCCAGGTTTGTAGTACCTCC	
*FASN*	Forward	TTCCGAGATTCCATCCTACG	122
	Reverse	AGGCTCACAAACGAATGGAC	
*GPAT1*	Forward	ATGGCATTCTTACAGTGGCAGAGC	183
	Reverse	GCTCCTTGGATTGGCTCACCTTC	
*PPARα*	Forward	ACGATTCGACTCAAGCTGGT	123
	Reverse	GTTGTGTGACATCCCGACAG	
*ACOX1*	Forward	GCCTCTGGATCTTCACTTGG	123
	Reverse	GTCTGGGCATAAGTGCCAAT	
*CPT1*	Forward	CAGACACCATCCAGCACATGAGAG	157
	Reverse	TGAGGCTCCGAGGTATTGTCCAG	
*CYP4A11*	Forward	TACATACAGGCCATTAGTGACC	83
	Reverse	CTGTAGATGGTGTCATTCTGGT	
*GAPDH*	Forward	TGGTGAAGACGCCAGTGGA	138
	Reverse	GCACCGTCAAGGCTGAGAAC	
